# A New Method for Calculating Femoral Anterior Cortex Point Location and Its Effect on Component Sizing and Placement

**DOI:** 10.1007/s11999-014-3930-1

**Published:** 2014-09-19

**Authors:** Mohamed R. Mahfouz, Emam ElHak Abdel Fatah, Lyndsay Bowers, Giles Scuderi

**Affiliations:** 1 Mechanical, Aerospace, and Biomedical Engineering Department, University of Tennessee, 307 Perkins Hall, Knoxville, TN 37996 USA; 2 Lenox Hill Hospital, North Shore LIJ Healthcare System, New York, NY USA

## Abstract

**Background:**

Variation in anterior femoral cortex morphology can cause improper component placement and alignment. When surgical inaccuracies occur, the mechanical properties of the distal femur may be altered, which could result in lower surgical success rates and an increased chance of postoperative complications.

**Questions/purposes:**

The purpose of the study is to come up with a reproducible computational algorithm to simulate what the surgeon does in the operating room. This method could help in surgical preplanning, patient-specific instruments, and implant design. From there, we evaluated (1) the angular difference between reference alignment axes; and (2) whether the location of the anterior cortex point and alignment axes had an effect on implant placement and amount of bone resected in seven implant systems.

**Methods:**

We analyzed 470 femurs from white and black individuals. Two points were defined using automatic three-dimensional landmarking: sizing point and femoral resection anterior cortex (FRAC) point. Alignment axes including the transepicondylar, posterior condylar, distal anatomical (DAA), and mechanical axes (MAs) were automatically calculated and used along with the resection point to define the anterior reference plane. Two mechanical axes were defined for the purpose of this study: MA-1 is a virtual construct used in navigated surgeries defined as the axis joining the center of the femoral head and the knee center and MA-2 was calculated as the axis joining the center of the femoral head and distal exit point of the DAA. Amounts of anterior, posterior, and distal resected bone were calculated along with the difference in orientation between the alignment axes.

**Results:**

The mean angular difference between transepicondylar axis and posterior condylar axis (PCA) was 5.44° ± 2.99°. All seven implant families showed more total bone resection on both the lateral and medial sides when the implants were aligned using MA-2 and PCA+3 of external rotation (PCA+3) when compared with using MA-1 and PCA+3 (p < 0.01). Using MA-2 and PCA+3 as an alignment method reduced the amount of bone resection on both medial and lateral anterior surfaces from 1 to 2 mm.

**Conclusion:**

The FRAC point is a key landmark in the placement and sizing of the femoral component. Improper sizing, notching, undercutting, or overstuffing can occur based on selecting the highest or lowest cortex point.

**Clinical Relevance:**

Balanced placement, prevention of notching, and anterior and posterior cut balancing were accomplished when using the suggested cortex point.

## Introduction

Achieving accurate implant positioning in TKA is critically important. Modern surgical techniques help reduce the amount of variation shown through conventional cutting techniques; however, studies have shown that even experts in the field can show variation in implant sizing and positioning [12, 14, 16].

Anterior and posterior referencing systems are used to obtain correct component alignment while avoiding notching of the anterior cortex of the femur. Studies have suggested a wide range of frequencies in terms of femoral notching in TKA [4, 17]. Notching of the anterior femoral cortex may be important because the occurrence of periprosthetic supracondylar fracture has been reported to range from 0.3% to 4.8% after TKA [2–5, 11, 16, 18–20], whereas both clinical and biomechanical studies have shown anterior notching to be present in 40% to 52% of reported fractures [3]. Inaccuracies in component sizing and placement can be strongly dependent on the anterior cortex of the femur [14], especially when using an anterior referencing system. However, the morphology of the anterior cortex of the femur has proven variable [15] and there is no agreement on how to best account for morphological variation during cutting techniques and component placement.

Hence, the purpose of the study is to come up with a reproducible computational algorithm to simulate what the surgeon does in the operating room. This method could help in surgical preplanning, patient-specific instruments, and implant design. From there, we evaluated (1) the angular difference between different alignment axes; and (2) the effect of anterior cortex point and alignment axes on implant placement and amount of bone resected on seven implant systems.

## Materials and Methods

We analyzed 470 adult femurs (421 white [303 male, 118 female] and 49 black individuals [44 male, five female] with a mean age of 55 ± 18 years). All white individuals were of European descent. CT data sets were obtained through either the William M. Bass Donated Skeletal Collection in the Department of Anthropology or cadaver scans in the Center for Musculoskeletal Research, both at the University of Tennessee (Knoxville, TN, USA). Only normal, nonpathologic bones were included in this analysis; those with any abnormalities (including fractures, extreme varus/valgus > 10°, osteophytes, and osteoarthritis) were specifically excluded. CT data sets were acquired with 0.625- × 0.625- × 0.625-mm cubic voxels. DICOM images were manually segmented and surface models were generated. This segmentation process has been proven reliable with an interobserver error of 0.163 mm, intraobserver error of 0.105 mm, and pairwise interobserver variability of 0.269 mm [9]. We analyzed seven implant systems: Persona™ (Zimmer Inc, Warsaw, IN, USA), NexGen^®^ (Zimmer Inc), Natural-Knee^®^ (Zimmer Inc), Vanguard^®^ (Biomet, Inc, Warsaw, IN, USA), Sigma^®^ (DePuy Orthopaedics, Warsaw, IN, USA), Genesis™ II (Smith & Nephew, Inc, Memphis, TN, USA), and Triathlon^®^ (Stryker Orthopaedics, Mahwah, NJ, USA).

Segmented models for each femur were added to the bone atlas. In short, a bone atlas is a method for automatically generating a homologous point distribution along the entire bone surface across a population. This allows for automatic calculation of relevant surgical and anatomical landmarks on virtual models. For example, loci of the patellar groove can be localized on the atlas template bone and then automatically propagated across the bones in the data set [6–9].

**Fig. 1:**
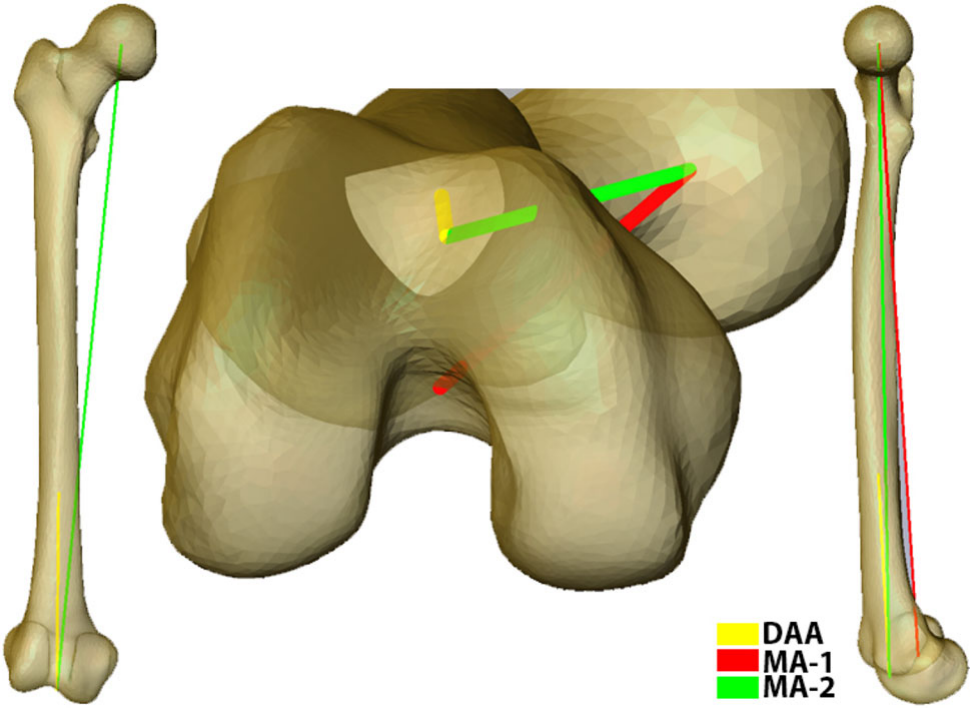
Diagram showing the calculation of the DAA (midline of the femoral shaft distal one-third) and two mechanical axes (MA-1 = line connecting femoral head center and knee center; MA-2 = line connecting femoral head center and distal exit point of the DAA).

Once the models have been added to the atlas and point correspondence is achieved [8], a set of anatomic and surgical landmarks was automatically calculated. Femoral landmarks include the medial and lateral anterior, posterior, and distal points; distal resection point; knee center; transepicondylar axis (TEA); posterior condylar axis (PCA); anatomic axis; and distal condylar axis [6, 8, 9]. The mechanical axis (MA-1) is a virtual construct used in navigated surgeries: defined as the axis joining the center of the femoral head and the knee center (intersection of the patellar groove plane and TEA) [8]. The distal anatomic axis (DAA) was defined as the axis approximating the distal one-third of the femoral shaft (ie, simulating an intramedullary guide). A second mechanical axis (MA-2) was calculated as the axis joining the center of the femoral head and distal exit point of the DAA (Fig. 1). To accommodate for the absence of cartilage, an average thickness of 1.8 mm was added to the distal aspect of the femoral condyle. The plane of distal resection was placed 90° to the mechanical axis and 10 mm proximal to the most distal part of the femur.

Two anterior cortex points were computed. First, the sizing point was defined as the saddle point, where the curve of the femur changes from convex to concave. This replicated the normal surgical practice of using a sizing guide. Second, the femoral resection anterior cortex (FRAC) point was defined as the point between the lowest and highest point on the lateral ridge that generates the most complete anterior resection contour (ie, prevents notching) and restores a balanced AP cut.

**Fig. 2:**
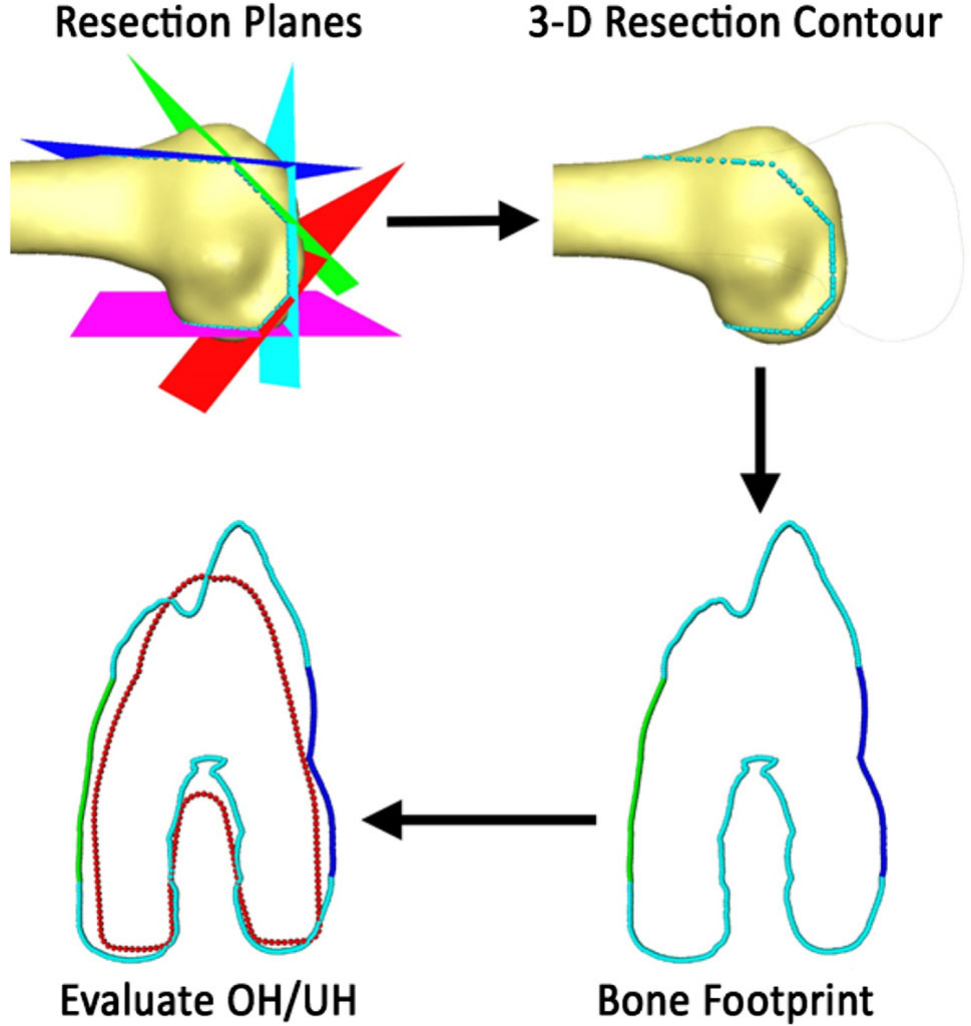
Evaluation of overhang and underhang was done using the bone and implant footprint. First, the 3-D resection profile is extracted, which is then flattened to generate the bone footprint. The generated footprint is then evaluated against the implant footprint to compute overhang (OH) and underhang (UH).

Implant sizing and placement for seven different designs were determined using landmarks. To find the proper implant size and placement, AP height was used to pick an initial implant size with the closest matching AP. The implant was placed using an anterior referencing technique with 0° internal/external rotation, 0° flexion/extension, and 0° varus/valgus. This method was selected to create a computer model of standard surgical practice. For component placement, we used three different longitudinal axes (MA-1, MA-2, and DAA) and three rotational measurements (PCA, PCA+3° of external rotation [PCA+3], and TEA). Final placement was performed using PCA+3 and MA-1. On placing the component, bone models were intersected with the implant cutting planes and a three-dimensional (3-D) contour was extracted, which was then flattened and used to calculate the bone footprint [8]. The bone footprint was then evaluated against the implant footprint to calculate overhang and underhang in the mediolateral direction (Fig. 2). Thresholds of 1.5 mm overhang and 2 mm underhang were used to refine the implant size. These values were selected to simulate reasonable threshold allowances without compromising surgical outcome and to further refine our computer model of real-life surgical practice [10]. The effect of using the computed FRAC point and the average point between the highest and lowest points on the lateral ridge was investigated. The resected bone thicknesses were measured on the anterior and posterior aspects of the femur for both the medial and lateral sides for each implant family. The four measurements included the anterior medial, anterior lateral, posterior medial, and posterior lateral thickness. The following angles between different alignments axes were computed to investigate the difference in axis alignment: (1) angle between PCA and TEA; (2) flexion/extension angle between MA-1 and DAA; (3) varus/valgus angle between MA-1 and DAA; (4) varus/valgus angle between MA-1 and MA-2; and (5) flexion/extension angle between MA-1 and MA-2.

## Results

**Fig. 3:**
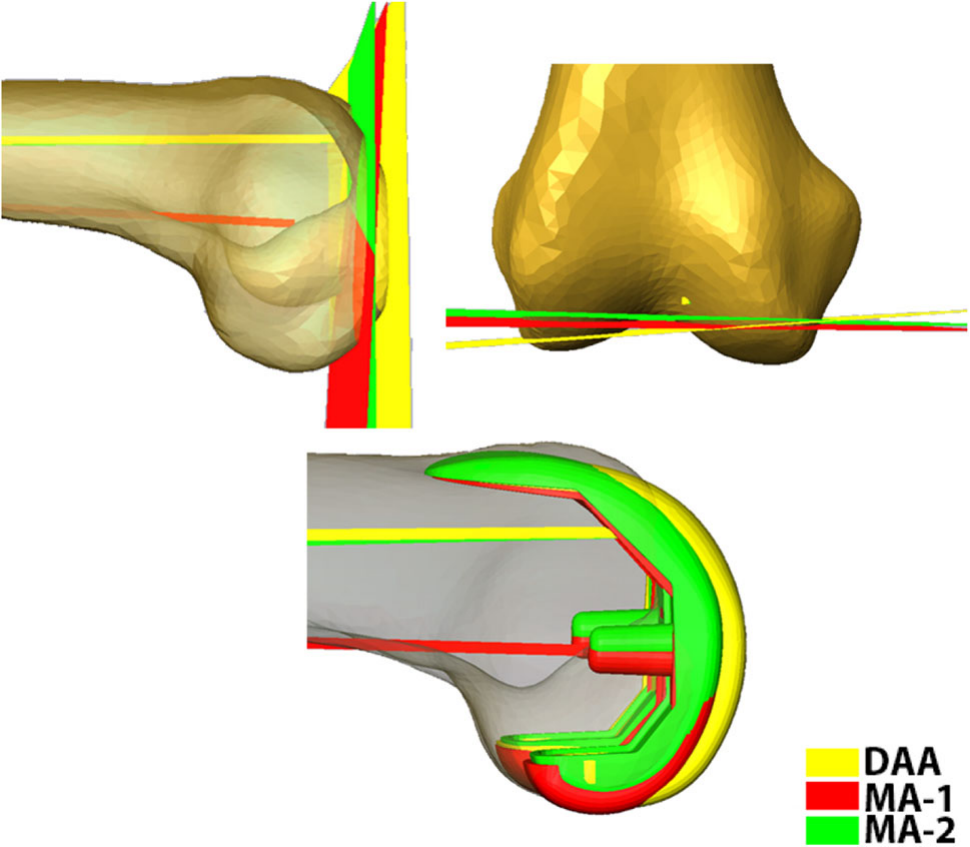
Differences in distal resection plane and implant placement resulted from the effect of using different axes (MA-1, MA-2, DDA) as alignment axes.

The mean of the 3-D angle between the PCA and TEA was 5.44° ± 2.99°. MA-1 showed 1.63° ± 0.82° (p < 0.01) more flexion and 5.14° ± 0.90° (p < 0.01) less valgus than the DAA (Table 1). MA-2 was 2.90° ± 0.38° (p < 0.01) more flexed than MA-1; the difference in varus-valgus was 0.23° ± 0.18°. These variations in axis selection resulted in different component alignment (Fig. 3).

**Table 1:** Angular differences between alignment axes

Angle	Angular difference (°)	
	Mean	SD
PCA TEA	5.44	2.99
MA-1 DAA FE	1.63	0.82
MA-1 DAA VV	5.14	0.90
MA-1 MA-2 FE	2.90	0.38
MA-1 MA-2 VV	0.23	0.18

PCA TEA = angle between PCA and TEA; MA-1 DAA FE = flexion/extension angle between MA-1 and DAA; MA-1 DAA VV = varus/valgus angle between MA-1 and DAA; MA-1 MA-2 FE = varus/valgus angle between MA-1 and MA-2; MA-1 MA-2 VV = flexion/extension angle between MA-1 and MA-2; PCA = posterior condylar axis; TEA = transepicondylar axis; MA-1 = mechanical axis 1 (the axis joining the center of the femoral head and the knee center); DAA = distal anatomic axis (the axis approximating the distal one-third of the femoral shaft); MA-2 = mechanical axis 2 (the axis joining the center of the femoral head and distal exit point of the DAA).

**Fig. 4A–B:**
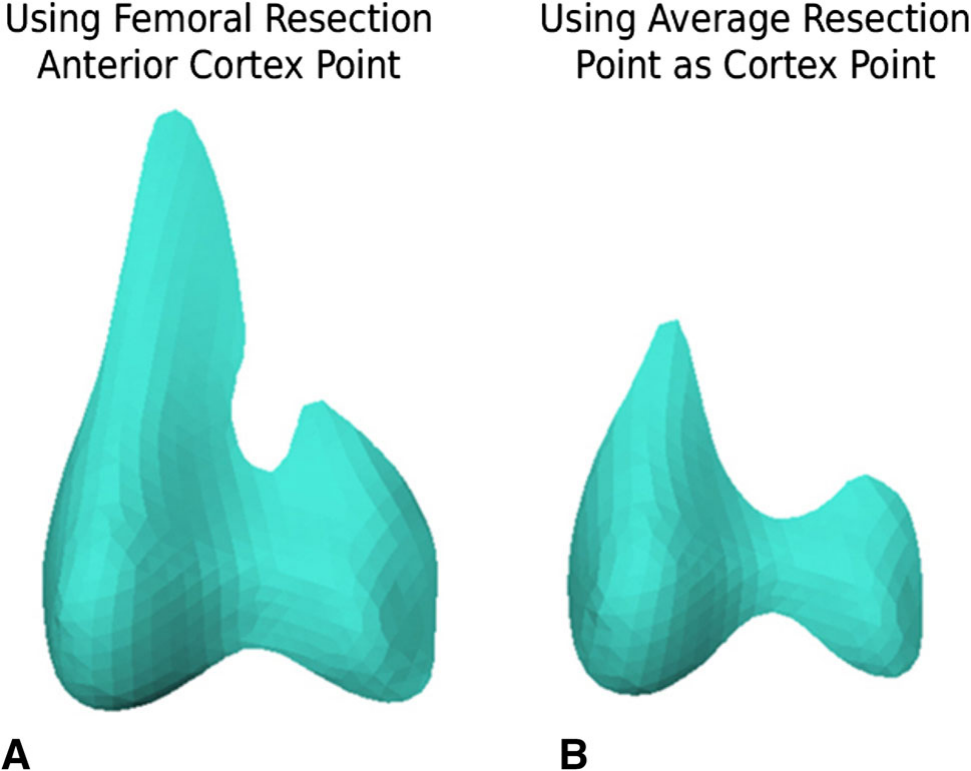
Difference in generated anterior resection profile using the FRAC point (**A**) and average point between highest and lowest lateral ridge points (**B**) is demonstrated.

Using the computed resection cortex point resulted in a more complete and balanced anterior resection surface (Fig. 4A) as compared with an undercut anterior resection surface when using the average point (Fig. 4B). All seven implant families showed more total bone resection on both the lateral and medial sides when the implants were aligned using MA-2 and PCA+3 when compared with using MA-1 and PCA+3 (p < 0.01) (Tables 2, 3). Using MA-2 and PCA+3 as an alignment method reduced the amount of bone resection on both medial and lateral anterior surface from 1 to 2 mm. However, the bone resection on the posterior surface increased from 3 to 5 mm medially or laterally depending on the implant, thus resulting in an increased total amount of bone resection with a range of 1 to 3 mm (p < 0.01). There was no statistical significance when comparing the average distal cut thickness (Table 4). The mean difference between medial and lateral distal resection was less than 0.25 mm.

**Table 2:** Amount of resected bone in various implants using MA-1 and PCA+3 as alignment axes

Variable	Amount of resected bone (mm)													
	Persona™		NexGen^®^		Natural-Knee^®^		Genesis™ II		Sigma^®^		Triathlon^®^		Vanguard^®^	
	Mean	SD	Mean	SD	Mean	SD	Mean	SD	Mean	SD	Mean	SD	Mean	SD
FACL	11	2	11	2	10	2	11	2	12	2	12	2	11	2
FPCL	8	2	12	3	13	3	10	3	7	3	7	2	10	2
Total lateral	19		23		23		21		19		19		21	
FACM	6	2	6	2	6	2	5	2	7	2	7	2	5	2
FPCM	11	2	14	3	16	3	13	3	10	3	10	2	12	2
Total medial	17		20		22		18		17		17		17	

MA-1 = mechanical axis 1 (the axis joining the center of the femoral head and the knee center); PCA+3 = posterior condylar axis + 3° of external rotation; FACL = amount of anterior lateral resected bone; FPCL = amount of posterior lateral resected bone; FACM = amount of anterior medial resected bone; FPCM = amount of posterior medial resected bone.

**Table 3:** Amount of resected bone in various implants using MA-2 and PCA+3 as alignment axes

Variable	Amount of resected bone (mm)													
	Persona™		NexGen^®^		Natural-Knee^®^		Genesis™ II		Sigma^®^		Triathlon^®^		Vanguard^®^	
	Mean	SD	Mean	SD	Mean	SD	Mean	SD	Mean	SD	Mean	SD	Mean	SD
FACL	10	2	10	3	9	2	9	3	10	3	10	3	9	3
FPCL	11	2	16	4	16	3	15	4	11	3	10	3	13	3
Total lateral	21		26		25		24		21		20		22	
FACM	5	2	4	2	4	2	4	2	5	2	5	2	4	2
FPCM	14	2	18	4	19	3	18	4	14	3	13	3	15	3
Total medial	19		22		23		22		19		18		19	

MA-2 = mechanical axis 2 (the axis joining the center of the femoral head and distal exit point of the distal anatomic axis); PCA+3 = posterior condylar axis + 3° of external rotation; FACL = amount of anterior lateral resected bone; FPCL = amount of posterior lateral resected bone; FACM = amount of anterior medial resected bone; FPCM = amount of posterior medial resected bone.

**Table 4:** Comparison of amount distal resected bone using MA-1 and PCA+3 and MA-2 and PCA+3 as alignment axes

Axes	Mean amount of resected bone (mm)	
	FDCL	FDCM
MA-1/PCA+3	5.01	8.90
MA-2/PCA+3	5.25	8.88

MA-1 = mechanical axis 1 (the axis joining the center of the femoral head and the knee center); PCA+3 = posterior condylar axis + 3° of external rotation; MA-2 = mechanical axis 2 (the axis joining the center of the femoral head and distal exit point of the distal anatomic axis); FDCL = amount of distal lateral resected bone; FDCM = amount of distal medial resected bone.

## Discussion

The success of TKA is dependent on accurate implant positioning and alignment. Continual advancements in technology and surgical techniques aim to reduce postoperative complications resulting from variations in component positioning. In this article, we introduce a new computational method for identifying the anterior cortex point. This technique produces an accurate and easily reproducible method reducing any variability that may occur in the operating room as a result of surgical technique, which can be used in component placement for preoperative planning, patient-specific instruments, or implant design. The study aimed to evaluate (1) the angular difference between different alignment axes; and (2) the effect of anterior cortex point and alignment axes on implant placement and amount of bone resected on seven implant systems.

There are a number of limitations to our study. First, the bone models used in this study are from cadaveric CT scans, which did not include the hyaline cartilage. This can affect the location of the distal resection plane; however, an offset of 1.8 mm from the bony distal surface to the distal resection plane reference point was included to account for this absence. This offset simulates the average cartilage thickness at the distal aspect of the femoral condyle. Second, the use of nonpathologic specimens may not reflect clinical reality and the degree of cartilage loss inherent in a clinical setting may result in variations of the posterior condylar axis and the distal resection plane. Still, this has no direct effect on the location of the FRAC point. Third, the method proposed in this study is a computational virtual method and cannot be implemented using conventional surgical techniques. However, the analysis can be performed preoperatively and translated into the operating room using either navigation or patient-specific instruments.

**Fig. 5:**
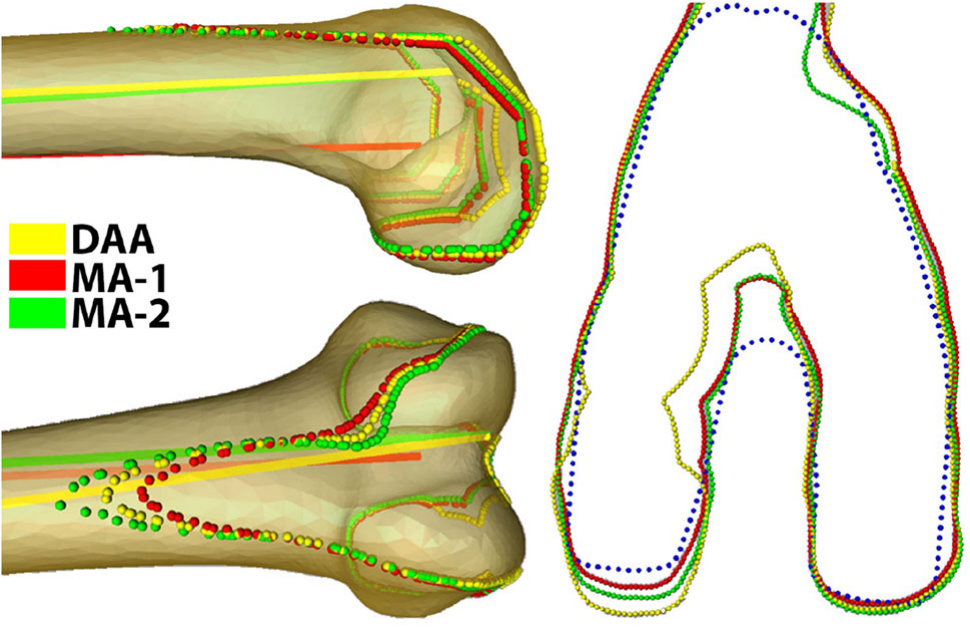
The effect of using different axes (MA-1, MA-2, DDA) as alignment axes on the generated footprint and the difference in flexion/extension and varus/valgus among the three axes is demonstrated.

The orientation of the femoral component is determined using different surgical alignment axes. The femoral component can be internally/externally aligned along the TEA, PCA, or PCA+3 depending on the surgeon’s preference and surgical technique being used. We measured the angular difference between the PCA and the clinical TEA to be 5.44°. This measurement is comparable to the results of Aglietti et al. [1] who reported an average difference of 5.6° between the TEA and PCA in varus knees. Restoration of the mechanical axis is a crucial step in component placement and has a direct impact on the outcome of TKA. Different techniques are used to calculate the MA. We investigated the use of the DAA as an alignment axis along with the use of MA-1 and MA-2. The varus/valgus angle between DAA and MA-1 was found to be 5.14°. This coincides with the results of Nam et al. [13] who reported a 5° difference between the mechanical and anatomic axis of the femur from radiographs. We found almost no (0.23°) varus/valgus difference between MA-1 and MA-2; however, the flexion/extension angle difference between MA-1 and MA-2 was found to be 2.9°. In general, using the DAA as an alignment axis can result in extreme valgus placement of the component, which directly affects the shape of the resection and generated footprint. We have found that using the DAA and MA-2 for implant alignment can lead to placement of the component in extension and thus cause notching resulting in a different footprint shape (Fig. 5).

Although anterior referencing techniques are used to obtain optimal component alignment while minimizing notching of the anterior cortex of the femur, studies have reported the frequency of anterior cortex notching to be as high as 41% [4, 17]. In this study, we defined the anterior reference plane to be in the same level as the FRAC point because locating the correct cortex point is a crucial step to prevent notching. Ng et al. [14] showed that femoral sizing varied depending on which of the nine measurement points on the anterior aspect of the femur were used as a reference. We defined the FRAC point as the point between the highest and lowest points on the lateral ridge, which minimized both notching and undercut. Anterior undercut would result when using the highest point on the lateral ridge, whereas notching of the anterior cortex would occur if using the lowest point. However, using an average point does not guarantee that notching is prevented or undercutting is minimized.

In an anterior reference TKA approach, preserving the shape of the anterior femoral cortex is important to prevent both patella-femoral joint overstuffing and femur notching. In this article, we proposed a computational method for calculating the femoral resection anterior cortex point to best preserve the shape of the anterior femoral cortex. Although the calculation of the proposed cortex point relies on digital models, these techniques can be translated to the operating room using either patient-specific guides or navigation.
